# Investigating the association of alerts from a national mortality surveillance system with subsequent hospital mortality in England: an interrupted time series analysis

**DOI:** 10.1136/bmjqs-2017-007495

**Published:** 2018-05-04

**Authors:** Elizabeth Cecil, Alex Bottle, Aneez Esmail, Samantha Wilkinson, Charles Vincent, Paul P Aylin

**Affiliations:** 1 Primary Care and Public Health, Imperial College London, London, UK; 2 Health Services Research & Primary Care, University of Manchester, Manchester, UK; 3 London School of Hygiene & Tropical Medicine, London, UK; 4 Medical Science Division, University of Oxford, London, Oxfordshire, UK

**Keywords:** health services research, statistical process control, healthcare quality improvement

## Abstract

**Objective:**

To investigate the association between alerts from a national hospital mortality surveillance system and subsequent trends in relative risk of mortality.

**Background:**

There is increasing interest in performance monitoring in the NHS. Since 2007, Imperial College London has generated monthly mortality alerts, based on statistical process control charts and using routinely collected hospital administrative data, for all English acute NHS hospital trusts. The impact of this system has not yet been studied.

**Methods:**

We investigated alerts sent to Acute National Health Service hospital trusts in England in 2011–2013. We examined risk-adjusted mortality (relative risk) for all monitored diagnosis and procedure groups at a hospital trust level for 12 months prior to an alert and 23 months post alert. We used an interrupted time series design with a 9-month lag to estimate a trend prior to a mortality alert and the change in trend after, using generalised estimating equations.

**Results:**

On average there was a 5% monthly increase in relative risk of mortality during the 12 months prior to an alert (95% CI 4% to 5%). Mortality risk fell, on average by 61% (95% CI 56% to 65%), during the 9-month period immediately following an alert, then levelled to a slow decline, reaching on average the level of expected mortality within 18 months of the alert.

**Conclusions:**

Our results suggest an association between an alert notification and a reduction in the risk of mortality, although with less lag time than expected. It is difficult to determine any causal association. A proportion of alerts may be triggered by random variation alone and subsequent falls could simply reflect regression to the mean. Findings could also indicate that some hospitals are monitoring their own mortality statistics or other performance information, taking action prior to alert notification.

## Introduction

The origins of the Imperial College Mortality Surveillance System lie in analyses commissioned by the Bristol Royal Infirmary Inquiry in 1999 examining paediatric cardiac surgical outcomes at the hospital. Our group confirmed serious concerns around the surgical outcomes at Bristol^1^ and established the usefulness of routine hospital administrative data in helping to identify quality of care issues. The current system alerts hospitals to high mortality rates in specific diagnosis and procedure groups by applying log-likelihood cumulative sum (CUSUM) charts^2^ to routinely collected hospital administrative data.^3^ The system has generated monthly mortality alerts on 122 diagnoses and procedures, for all English acute non-specialist National Health Service (NHS) hospital trusts, since 2007 (an acute trust is a single or group of hospitals which provide secondary health services, including emergency services, within the English NHS). Shortly after commencing, the mortality alerting system was critical in triggering the initial investigation into Stafford Hospital, which highlighted severe failings in emergency care and led to a series of enquiries culminating in the Mid Staffordshire NHS Foundation Trust Public Inquiry led by Sir Robert Francis.^4^

The alerts, highlighting potential problems, are individually assessed and then sent out to the alerting trusts. An example of an alert letter is shown in online supplementary file 1. Hospitals are notified within 3 months of an alert being triggered^3^ (table 1). On receiving an alert, the hospital trust will have an opportunity to carry out its own internal investigation, which may involve an examination of coding, and often will include a case note review. The trust will formulate an action plan if deemed necessary.

10.1136/bmjqs-2017-007495.supp1 Supplementary data

**Table 1:** Timings of alert, letter and investigation by the Care Quality Commission (CQC) to assess the actions of an alerting hospital

Time in months, median (range)	Events
0	Cumulative risk-adjusted mortality rates within a hospital for a given condition or procedure exceed a set threshold.
3 (3–4)	Mortality surveillance and alerting system, using administrative hospital inpatient data, detects the high mortality and triggers an alert. The alerting hospital and the CQC are notified by letter.
6 (3–7)	The CQC assesses information it holds on the alerting hospital, and opens a case usually requesting (1) evidence of case note audit of the relevant patient groups and (2) evidence of actions taken to make improvements.
9 (6–14)	The CQC investigations are completed (1) closing the case as the CQC is satisfied with the hospital’s actions or (2) referring the case for further investigations with local and regional teams.

The Imperial Unit also notifies the Care Quality Commission (CQC) of all alerts generated by the system. The CQC, as the regulator of health and social care in England, is a key stakeholder in the use of hospital data for performance monitoring. The CQC follow up alerting trusts, commonly requesting evidence of clinical audit of the relevant patient groups and evidence of actions taken to make improvements. There is little information available on individual hospital responses to a mortality alert. However, an independent audit of CQC data, carried out by our team,^5^ found that CQC investigations, which required appropriate actions from the alerting trusts, were completed on average within 9 months of the alert being triggered (table 1). The CQC also runs its own alerting system, the CQC outliers programme, which is based on similar methods to the Imperial College system, but differs in risk adjustment.

Investigating healthcare performance is a complicated and potentially expensive activity, so it is important to assess whether the monitoring is associated with improved performance. Our aim was to investigate the association between a mortality alert letter and subsequent mortality rates, in acute NHS hospital trusts in England. Our hypothesis was that the alerting letters highlight areas of concern, and alongside CQC investigations lead to improvements in care which ultimately impact on patient outcomes such as mortality. The pattern of alerts, the actions taken by the CQC, and the relationship between mortality alerts and other indicators of quality of care are described in other publications arising from an National Institute for Health Research-funded project to examine the impact of a national mortality alerting system.^5^

## Methods

We investigated the association between a mortality alert and subsequent mortality using an interrupted time series (ITS) design. ITS is a strong, quasi-experimental approach for evaluating longitudinal effects of interventions.^6^

### Setting and participants

Our setting was acute non-specialist NHS hospital trusts in England (around 135 trusts—the number monitored by the Imperial Unit fluctuates due to mergers, closures and new hospitals opening). The participants were those trusts that had received notification of a mortality alert generated between January 2011 and November 2013.

### Intervention

An alert indicates sustained higher than expected in-hospital mortality in 1 of 122 diagnosis/procedure groups. The alerts are generated by applying log-likelihood CUSUM charts^2^ to the most current Hospital Episode Statistics (HES) data available and plotting the individual-level difference, for a specific diagnosis or procedure, between the actual outcome (patient death) and the case mix-adjusted predicted risk of mortality.^3^ HES, the national administrative database, holds details of inpatient activity from all acute NHS hospital trusts in England. Each record within the HES contains data on patient demographics such as age, ethnicity and socioeconomic deprivation based on postcode of residence; the episode of care such as hospital name, date of admission, date of discharge and discharge destination, which includes a code for death; and clinical information.^7^ Within the HES, the main reason for an admission or ‘primary diagnosis’ is recorded using the International Classification of Diseases, 10th revision (ICD10) codes. The ICD10 codes were mapped to clinically meaningful categories (or diagnosis groups) using the Agency for Healthcare Research and Quality Clinical Classifications System.^8^ Procedure code groupings were based around OPCS4 codes (see online supplementary file 2 for a list of procedure and diagnosis groups). After sustained higher than expected mortality over time, a preset threshold is crossed, triggering a mortality alert. When developing the tool, the emphasis was on suppressing the false alarm rate, as a large number of false alarms would erode confidence in using the tool.^3^ The threshold, set at a level to ensure an estimated false alarm rate of 0.1% over a 12-month period of monitoring, is tailored to each hospital by taking into account the annual number of admissions for the diagnosis or procedure group at the hospital.^9^ Not all alerts are sent out. Each alert generated is reviewed individually by the Imperial Unit, and alerts are withheld if they represent small numbers of deaths (fewer than five expected deaths) or they are repeat signals (within 9 months) for which the hospital has already been alerted.

10.1136/bmjqs-2017-007495.supp2 Supplementary data

### Outcome

**Figure 1:**
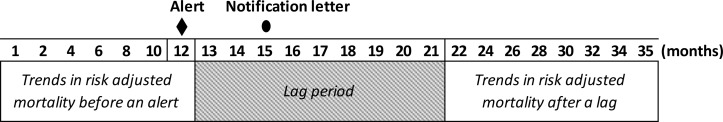
Timing in months before an alert, the alert, the notification, lag and post lag.

Our outcome was the monthly relative risk of in-hospital mortality by hospital trust and diagnosis/procedure group. This was derived from the final annual extract of the HES. A trust-level relative risk was calculated for each diagnosis/procedure group and trust based on the sum of observed deaths per month divided by expected deaths. Expected deaths were based on the probability of death for each individual patient. The probability is estimated using a logistic model and includes the patient’s age, sex, diagnosis subgroup, emergency/elective admission, month of admission, Charlson Comorbidity Index,^10^ Carstairs Socioeconomic Index,^11^ the number of previous emergency admissions and seasonality. We calculated monthly relative risk of mortality for February 2010 until December 2014. For each alert, there was a 35 months’ follow-up of diagnosis/procedure group-specific outcome data: 12 months prior to an alert (including the month of the alert) and 23 months post alert (figure 1). Any repeat alerts (for the same trust and diagnosis/procedure group) during the 23 months postalert follow-up were excluded (online supplementary file 2).

### ITS statistical analysis

We investigated trends in relative risk before an alert and following the alert (with a lag period) using segmented regression. We expected that there would be a 9-month lag before any changes in trends would occur. This is an anticipated time, estimated from our team’s study into the CQC investigations of these alerts^5^ (table 1), for a hospital trust to receive the mortality alert letter, and to investigate potential causes and effect change within the hospital setting.

Our model measured three parameters—the increasing slope in relative risk prior to an alert, a level (step) change after a 9-month lag period and the slope following the lag period (over 14 months). Data over the lag period were left out of the model. We chose to analyse the 12 months up to an alert and 14 months after the lag believing that this was sufficient time to robustly model the pre-existing and postlag trends.

We modelled the data (observed offset by the expected number of deaths) using generalised estimating equations (GEE) based on a Poisson distribution. This semiparametric modelling compensates for the correlation between repeated measures of relative risk from individual hospital trusts over the study period. It also allows for distribution assumptions of the data to be relaxed. Similar methodology has been assessed against a randomised controlled trial.^12^ GEE calculates population-averaged parameter estimates. We clustered by trust and diagnosis/procedure group. We applied an exchangeable correlation matrix. ITS is a statistical investigation that allows to adjust for trends in the outcome over time and can be a strong tool for investigating interventions. However, this methodology does have several assumptions^13^: (1) that the intervention occurred independently of other changes over time; (2) the intervention was unlikely to affect data collection; (3) the outcome was reliable; and that (4) we have appropriately adjusted for autocorrelation. Autocorrelation is a serial correlation and refers to the relationship between an outcome’s current value and its past values. We corrected for autocorrelation by incorporating a delay variable in our model, which was defined as the difference between the observed and predicted value of the dependent variable (in this case relative risk) from the previous observation. We modelled all alerts but also subsets of two diagnosis/procedure groups—acute myocardial infarction (AMI) and sepsis. These groups were selected a priori as they commonly contributed to mortality alerts.^14^

To try to fully characterise trends in mortality following an alert, we carried out a sensitivity analysis to investigate trends with reduced lag time (0, 3, 6 months). We also investigated crude risk of death, the observed deaths divided by the number of admissions.

We estimated the number of alerts expected through chance alone (statistical false alarms) by the number of hospital trusts monitored × number of diagnosis/procedure groups monitored × annual false alarm rate (based on our predefined threshold of 1 per 1000, which takes into account the annual number of patients at each trust and the outcome rate for each patient group).^9^

### Patient involvement

This paper is part of a larger project evaluating a national surveillance system for mortality alerts. There were two patient representative members of the Scientific Advisory Group who contributed to the development of the research question and outcomes of this study. There was a consultation with the members of the public through peopleinresearch.org, and five participants attended a focus group which discussed mortality alerts and the justification for using personal data to generate them.

## Results

Two hundred and fifty five alerts were generated between January 2011 and November 2013, of which 203 were sent out to hospital trusts. Thirty-one of the sent alerts were repeat alerts or had insufficient follow-up due to hospital closures/mergers (online supplementary file 2). We analysed 172 alerts sent to 93 acute NHS hospital trusts in England. Of these, 8 were for AMI and 19 for sepsis. Sepsis was the most commonly alerting diagnosis/procedure, followed by liver disease (alcohol-related) (10 alerts) (online supplementary file 2). The total number of deaths in the alerting hospitals was 14 452 over the study period compared with 11 083 expected deaths in a total of 266 468 admissions. The mean number of monthly deaths (by trust and diagnosis/procedure group) rose in the 12 months prior to and fell directly after an alert, while the number of admissions and expected mortality remained constant over the course of the study (table 2 and online supplementary file 2).

**Table 2:** Mean monthly number of admissions, observed and expected deaths, by follow-up time (in quarters)

Time in months	Admissions	Observed deaths	Expected deaths
	Mean (95% CI)	Mean (95% CI)	Mean (95% CI)
Prealert			
1–3	44.8 (38.4 to 51.1)	2.42 (2.20 to 2.64)	1.86 (1.68 to 2.03)
4–6	46.0 (39.4 to 52.6)	2.64 (2.41 to 2.86)	1.88 (1.71 to 2.05)
7–9	46.1 (39.7 to 52.5)	3.00 (2.73 to 3.27)	1.93 (1.74 to 2.12)
10–12	46.2 (39.8 to 52.6)	3.77 (3.43 to 4.10)	1.99 (1.80 to 2.18)
Lag period			
13–15	46.7 (40.2 to 53.3)	2.49 (2.23 to 2.75)	1.93 (1.75 to 2.11)
16–18	46.5 (39.9 to 53.1)	2.21 (1.97 to 2.45)	1.81 (1.64 to 1.98)
19–21	46.9 (40.6 to 53.2)	2.37 (2.12 to 2.62)	1.96 (1.78 to 2.15)
Post lag			
22–24	46.2 (40.2 to 52.2)	2.20 (1.97 to 2.43)	1.93 (1.75 to 2.12)
25–27	46.6 (40.5 to 52.8)	2.26 (2.03 to 2.50)	1.94 (1.76 to 2.13)
27–29	45.4 (39.5 to 51.3)	2.07 (1.83 to 2.31)	1.93 (1.73 to 2.13)
30–33	44.6 (38.8 to 50.3)	2.22 (1.95 to 2.49)	1.93 (1.73 to 2.14)
34–35	44.7 (37.2 to 52.2)	1.89 (1.59 to 2.18)	1.79 (1.54 to 2.03)

Mean monthly statistics are calculated from individual trust, diagnosis/procedure group data. Observed and expected numbers of death are inpatient deaths. Expected deaths are estimated using case mix risk adjustment.

Our model estimated, on average, a monthly increase in relative risk of 5% (95% CI 4% to 5%) prior to an alert, representing 50% increase in the 12 months prior to an alert. There was a 61% fall during the 9-month lag period (95% CI 56% to 65%) and a continued reduction (non-statistically significant) of 1% per month (95% CI 0% to 2%) (table 3). On average, the risk of death reduced to an expected risk (ie, the relative risk returned to 1) within 18 months of an alert. Modelling crude risks also estimated falls in risk following an alert. There was a 75% fall in crude risk after a 9-month lag (95% CI 72% to 77%). Modelling mean observed deaths also displayed similar patterns (61% fall after a 9-month lag).

**Table 3:** Interrupted time series analysis modelling adjusted relative risk and crude monthly mortality for all alerts (diagnoses and procedures), AMI alerts and sepsis alerts

	All alerts n=172			AMI n=8			Sepsis n=19		
	Risk ratio	95% CI	Change (%)	Risk ratio	95% CI	Change (%)	Risk ratio	95% CI	Change (%)
Modelling adjusted risk									
Prealert trend	1.05	(1.04 to 1.05)	5***	1.05	(1.02 to 1.08)	5***	1.04	(1.02 to 1.06)	4***
Level change (after lag)	0.39	(0.35 to 0.44)	−61***	0.43	(0.28 to 0.67)	−57***	0.41	(0.29 to 0.59)	−59***
Postlag trend	0.99	(0.98 to 1.00)	−1	0.99	(0.94 to 1.04)	−1	1.00	(0.96 to 1.05)	0
Modelling crude risk									
Prealert trend	1.07	(1.06 to 1.07)	7***	1.08	(1.05 to 1.11)	8***	1.04	(1.03 to 1.06)	4***
Level change (after lag)	0.25	(0.23 to 0.28)	−75***	0.27	(0.18 to 0.41)	−73***	0.31	(0.24 to 0.39)	−69***
Postalert trend	0.99	(0.98 to 1.00)	−1	0.98	(0.95 to 1.01)	−2	0.99	(0.97 to 1.01)	−1

Risk ratios are the model estimated ratios of relative risk of death (observed number/expected number) in the adjusted model and the rate ratios of death rate (observed number/admission number) in the crude model. Trend risk ratios are monthly increases/decreases. Our model measures the trend prior to an alert, the level change during varying lag periods and postlag trend in relative risk of death. Models are adjusted for autocorrelation. The model uses generalised estimating equations and the Wald test statistical significance was ***p<0.001. The 172 alerts were generated between January 2011 and November 2013 and sent to 93 acute National Health Service trusts in England.

AMI, acute myocardial infarction.

**Figure 2:**
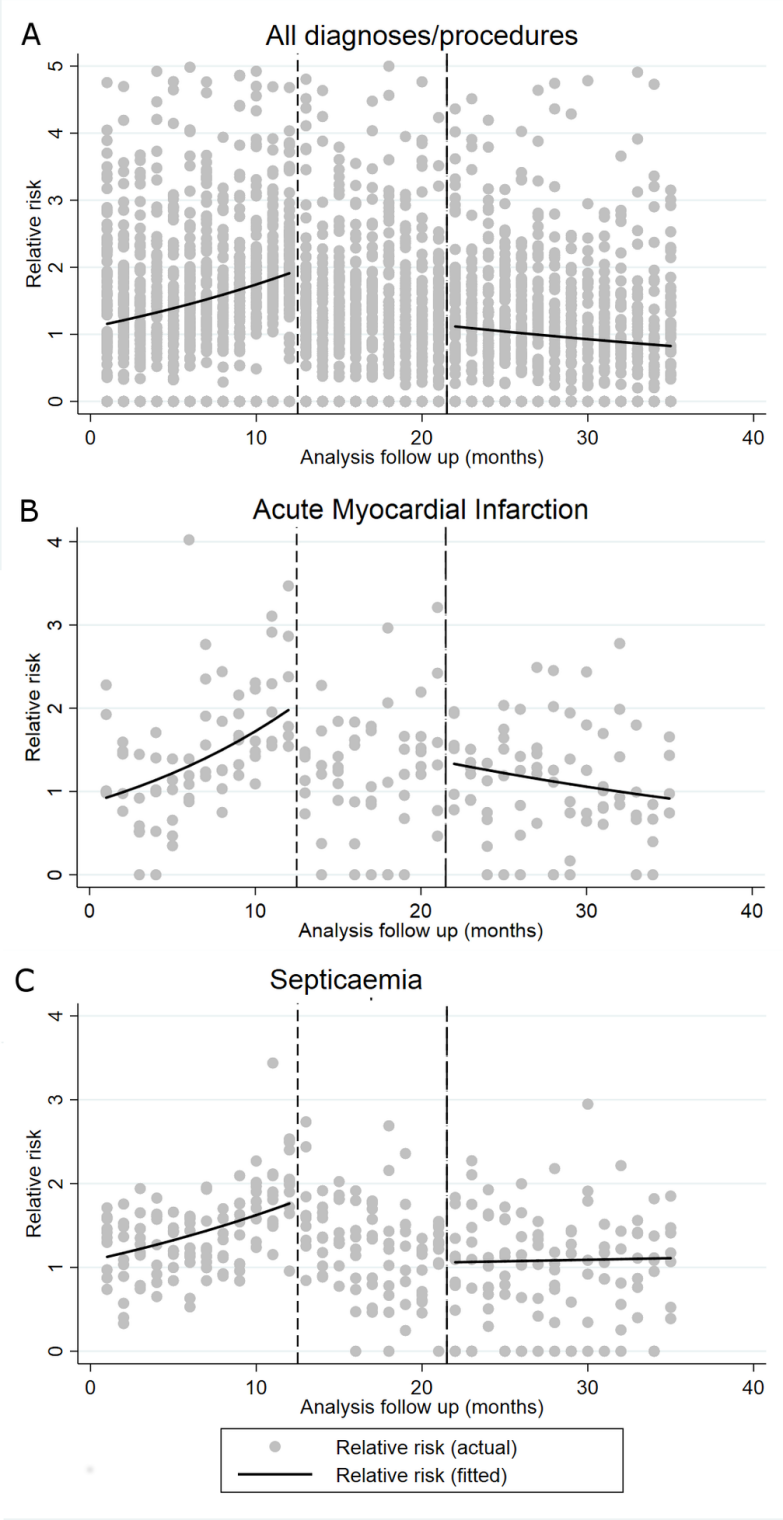
Trends in relative risk of death before a mortality alert and after postalert 9-month lag period for (A) all diagnosis/procedure groups (for values <5), (B) acute myocardial infarction and (C) sepsis.

The trends in relative risk of death before a mortality alert, during the 9-month lag period and after the lag period for all diagnoses/procedures, AMI and sepsis are displayed in figure 2.

Sensitivity analyses, with a reduced lag time, also estimated immediate falls in relative risk following an alert. We estimated a 38% (34%–42%), 46% (41%–50%) and 52% (47%–56%)%) fall with no, 3-month and 6-month lags, respectively (table 4).

**Table 4:** Interrupted time series analysis modelling adjusted monthly mortality risk with no, 3-month and 6-month lag periods

	All alerts n=172		
	Risk ratio	95% CI	Change (%)
Modelling with no lag			
Prealert trend	1.05	(1.04 to 1.05)	5***
Level change (over lag)	0.62	(0.58 to 0.66)	−38***
Postlag trend	0.99	(0.98 to 1.00)	−1
Modelling 3-month lag			
Prealert trend	1.05	(1.04 to 1.05)	5***
Level change	0.54	(0.50 to 0.59)	−46***
Postalert trend	0.99	(0.99 to 1.00)	−1
Modelling 6-month lag			
Prealert trend	1.05	(1.04 to 1.06)	5***
Level change	0.48	(0.44 to 0.53)	−52***
Postalert trend	0.99	(0.98 to 1.00)	−1

Risk ratios are the model-estimated ratios of relative risk of death (observed number/expected number). Trend risk ratios are monthly increases/decreases. Our model measures the trend prior to an alert, the level change during varying lag periods and postlag trend in relative risk of death. Models are adjusted for autocorrelation. The model uses generalised estimating equations and the Wald test statistical significance was ***p<0.001. The 172 alerts were generated between January 2011 and November 2013 and sent to 93 acute National Health Service trusts in England.

AMI and sepsis model-estimated parameters were similar to those for all diagnosis/procedure groups, showing immediate falls in relative risk following an alert, although small numbers made CIs wider (table 3).

Over the study period, we monitored 135 acute non-specialist trusts^15^ for 122 diagnosis/procedure groups. The annual probability of an individual diagnosis/procedure group in each trust in each year generating a statistical false alarm was 0.001 (0.1%), therefore potentially up to 17 alerts over a 12-month period of monitoring or 49 over 3 years. Therefore potentially 19% (49/255) of alerts generated over the study period may have been triggered by random variation alone.

## Discussion

Our study of a national mortality surveillance system and its association with subsequent trends in relative risk of mortality found that, on average, the risk of death fell by 61% in the 9 months following an alert, then declined more slowly, reaching the level of expected risk within 18 months of the alert.

### Findings in relation to other studies

Monitoring healthcare performance is common, and there are plenty of examples of evaluations of accreditations within healthcare providers which mainly focus on care processes.^16^ However, there are few studies that investigate the monitoring of a mortality surveillance programme, and the information held within these reports is limited.^17–19^ There is one other study that attempted to evaluate the impact of a monitoring (benchmarking) system on outcomes over time.^20^ This study, investigating surgical outcomes including in-hospital mortality, was limited. Data available for the analysis were only available from hospitals that participated in the programme and no data were available before the hospitals joined the programme. As a result, the study was unable to adjust for secular trends, and findings of a decreased in-hospital mortality over time may be unrelated to the intervention. Although there are no UK studies that focus on tools for monitoring mortality, a recent study investigated the association between the CQC’s Intelligent Monitoring statistical surveillance tool and the subsequent inspection-based quality ratings.^21^ It included all 103 inspections carried out in the 2 years following the launch of the Intelligent Monitoring statistical surveillance tool and concluded that the tool could not predict the outcomes of NHS hospital trust inspections. There was high variability in the outcome, and the authors point out that the surveillance tool, using a combination of 150 individual indicators to produce an unweighted, trust-level score, may be too coarse. Skilled inspectors are able to identify localised pockets of poor quality within a hospital trust which may be obscured, and previous studies have indicated that within-hospital variation in outcomes between hospital departments and specialties is high.^22 23^ As a result, the Imperial College Mortality Surveillance System, which focuses on a single outcome and is condition-specific, should be better at detecting localised quality of care issues.

### Limitations

Our study is the first evaluation of the impact of a national mortality surveillance system on subsequent hospital mortality, and the strengths of ITS have previously been reported and have been compared favourably with traditional clinical trial methodology.^12 24 25^ However, there are limitations to our study which need to be highlighted.

### Controls

We assume that the intervention occurred independently of other changes in time but did not control for this. A difference in difference model using internal or external controls would not have been appropriate. Alerts are not constrained to a single point in time so selecting a control from a non-alerting trust will not account for secular trends. Risk-adjusted mortality is likely to be diagnosis/procedure group-specific within a trust^22 23^; however, selecting a control from within the same trust by using a non-alerting diagnosis/procedure group would not clarify the issue either. A rise in risk-adjusted mortality could imply that there are external factors that are causing the effect; however, it also could imply issues associated with increased mortality were not confined to a single diagnosis/procedure group within the trust but were systemic.

### Risk models

The risk models underlying the system take into account a number of factors,^26^ but there may be other confounders which we were unable to adjust for, such as disease severity; however, there are unlikely to be sudden changes in disease severity coinciding with each of our alerts, therefore we doubt that changes in case mix could explain our findings.

It is possible that rebasing of the statistical model used to generate predicted risks contributed to the differences between adjusted and unadjusted models. Rebasing is the establishment each year of a new base level for case mix adjustment and adapts the risk adjustment using the latest 10 years of data. This is needed due to the long-term national trend of falling in-hospital mortality. We tested this effect on our ITS by including financial year as an explanatory variable, and found no effect.

### Data submission

HES data, provided monthly by the Health and Social Care Information Centre (HSCIC, now called NHS Digital),^27^ were used to generate the mortality alerts in this analysis. These monthly data are provisional. The HSCIC also produces a final annual extract which covers a financial year, and this annual snapshot of healthcare activity is used to estimate mortality trends in our analysis. The provisional and final extracts will differ since trusts can resubmit data following initial submission, resulting in changes to the data in the interim period.

### Coding

The CQC mandates yearly audits of individual hospital data quality, which will trigger an audit of coding within the hospital^28^; as a result, the coding of the primary diagnosis and procedure is of high accuracy,^29^ yet post-hoc changes to the data may be made following an alert, which could lead to shifts in diagnosis or changes to comorbidity coding, potentially leading to some change in crude and adjusted mortality. If this is the case, then this will violate our assumption that the intervention was unlikely to affect data collection.^13^ We investigated changes in coding after AMI and sepsis alerts and found that the majority of trusts appeared to have made some changes to their data after an alert.^5^ We found the average number of observed and expected deaths fell, while relative risk of death increased by only 1%–5% following changes in coding (see online supplementary file 2).^5^ These small increases could not account for the falls in relative risk we find, following an alert. If changes in comorbidity coding accounted for our observed fall, we would also expect effect sizes to be different in adjusted and unadjusted models. We found large falls in both crude and relative risk after a 9-month lag, although the magnitude of the fall in the unadjusted model was greater.

### Regression to the mean

The use of log-likelihood CUSUM charts and high thresholds limits the role that chance has to play in creating statistical false alarms. CUSUM charts, compared with other types of statistical control charts, give the greatest chance of detecting a true change in the outcome measure for a given false positive rate.^30^ The log-likelihood method of Steiner *et al* 2 includes adjustment for case mix. Even so, one in five of the Imperial mortality alerts could have been triggered by random variation alone and subsequent reductions due to regression to the mean. Other interventions to reduce hospital mortality have rarely been linked to such dramatic reductions.^31–33^

### Possible explanations and implications for clinicians and policymakers

Our models found that trust-level relative risks of death fell after an alert and a majority of the decrease took place within the lag period, the period we hypothesised that it would take for the trust to be notified by an alert letter and implement changes to reduce mortality. For example, there was a 38% fall in relative risk of death, the month after an alert and 52% fall after 6 months. Given these results and our estimate that around 20% of our alerts may be due to chance alone, it is difficult to discern any true effect of the surveillance system. Our findings could indicate that our lag period is wrong, and that hospitals are monitoring their own mortality statistics or other relevant performance information that correlates with mortality, and are taking action before receiving the alert letter. This is reflected in information gathered from site visits in a sample of alerting hospitals.^5^ However, we cannot discount the explanation that the observed reduction is a result of regression to the mean (the phenomenon that after extreme measurements, in this case resulting in a mortality alert, subsequent measurements are likely to fall).^34^ We may have underestimated the proportion of false alarms. Our estimate assumes all trusts (for all conditions/procedures monitored) have the same underlying mortality. There may, however, be variability between trusts, with some having higher underlying mortality than others; in these trusts chance variation is more likely to put the relative risk of death over the threshold required to cause an alert, potentially increasing the proportion of alerts that were false alarms. We also excluded alerts that occurred within a trust (for the same diagnosis/procedure group) during the follow-up period (33 months), which may also have introduced bias as these excluded alerts are more likely to be persistent signals.

Although there is some controversy in using hospital mortality to investigate quality of care, major concerns centre on summary measures of hospital mortality, such as the Hospital Standardised Mortality Ratio and the Summary Hospital-level Mortality Indicator.^35 36^ The system we investigate focuses on specific diagnosis and procedure groups, and a value to monitoring mortality in specific groups of patients has been recognised.^37^ Our qualitative research has found strong senior leadership support for mortality monitoring where alerts are considered a useful tool in providing a focus to help reduce mortality.^5^

### Unanswered questions and future research

While each hospital trust is expected to have a policy in place that sets out how it responds to the issue of potentially avoidable mortality,^38 39^ to date there has been little information on how trusts respond to mortality alerts.^40^ As part of this NIHR-funded project, we have gained further insight into institutional mortality monitoring and the response to alerts through indepth qualitative case studies within alerting hospitals. Although this work is detailed in the project report and will form the basis of future publications, it remains to be seen whether better quality and more clinically focused data could improve the validity of these systems. There is also a need to explore more fully the role of chance and regression to the mean in statistical process control-based surveillance systems through simulation.

## Conclusion

Mortality rates fell over the 9-month period following an alert and on average approached the expected rate at 18 months. The fall in mortality in many cases appeared to precede any reasonable time lag for action and it is difficult to infer any causal association from our analysis. A proportion of alerts may be triggered by random variation alone and subsequent falls could simply reflect regression to the mean. Findings could also reflect that hospitals are monitoring their own mortality statistics, taking action before our alert letter. There is a need to explore more fully the role of chance and regression to the mean in statistical process control-based surveillance systems through simulation.
