# Supplementary material for: Investigating the association of alerts from a national mortality surveillance system with subsequent hospital mortality in England: an interrupted time series analysis
Source: BMJ Qual Saf. 2018 May 4;27(12):965–73. doi: 10.1136/bmjqs-2017-007495 (PMC6288695; doi:10.1136/bmjqs-2017-007495)
Supplement: Supplementary data [file bmjqs-2017-007495supp001.pdf]

XXXXXX (name)
Chief Executive
XXXXX NHS Trust
XXXXX Hospital
XXXXXX (Address)
XXXXXX (City)
XXXXX (Postcode)

15 June 2016

Dear XXX,

MORTALITY OUTLIERS

We are writing to share with you in confidence an analysis of mortality data which indicates higher than average mortality rates for Intracranial injury within your hospital trust (Appendix 1).

The Dr Foster Unit at Imperial College (DFU) routinely analyses Hospital Episode Statistics (HES) data for a wide range of diagnoses and procedures, computing risk-adjusted mortality rates for hospitals. In the course of this work we have come across examples of mortality rates in various trusts significantly in excess of what would be expected, given the risk profile of the relevant patients.

There are a number of possible reasons for these results, including random variation, poor data quality or coding problems, and case-mix issues, and we draw no conclusions as to what lies behind the figures. However, as clinicians we believe we have a duty under the GMC Good Medical Practice code to alert trusts to this analysis since there is a possibility that it indicates areas where patients may be at risk.

We therefore piloted a system of mortality alerts to trusts in 2007 and received very valuable feedback. As a result of the pilot we have made a number of changes for the roll-out of the alert system. First, we have limited the procedures and diagnoses we monitor for the purposes of this alert system, as we wish to restrict alerts to areas where there is most likely to be a clinical issue. Second, we have increased the amount of information supplied with the alerts. The short briefing note at Appendix 2 explains our methodology and alert process in more detail, including the criteria we have used for alerting trusts.

Third, we have decided to share alerts routinely with the Care Quality Commission. The general view of the trusts we consulted in our pilot was that it was appropriate for the Commission to receive this information as part of the wide range of data it receives about individual trusts. The Commission has said that it will consider these alerts using its own internal analytical process and then decide whether or not they represent a concern in the context of all the other information it holds with respect to the trust. The Commission will follow these up by writing to you either asking for further information or to inform you that it has no current concerns.

The purpose of this alert system is, in essence, both to discharge our ethical duty and to provide trusts with information which we hope you will find helpful. What action you do or do not take is entirely a matter for you; although we would be very happy to receive feedback on this alert, please do not feel obliged to acknowledge this letter. We should add that we only have limited capacity to provide follow-up advice and analysis, although we are happy to answer specific technical queries.

Finally, we must stress that this is an initiative of the Dr Foster Unit at Imperial College, not Dr Foster. Information on which trusts we send alerts to has not and will not be shared with Dr Foster for marketing or other purposes.

Yours sincerely

Professor Sir Brian Jarman OBE MA PhD FRCP FRCGP FFPH FMedSci
Professor Paul Aylin MBChB FFPH

Department of Primary Care and Public Health
Imperial College London
Dr Foster Unit
3 Dorset Rise
Ground Floor
London
EC4Y 8EN

Appendix 1

Hospitals name

Intracranial injury
S06,T060,T904,T905,T908,T909

This chart indicates that on at least one occasion in the three months to Mar 2016, risk-adjusted mortality of double the expected rate was recorded at this trust for this diagnosis or procedure.

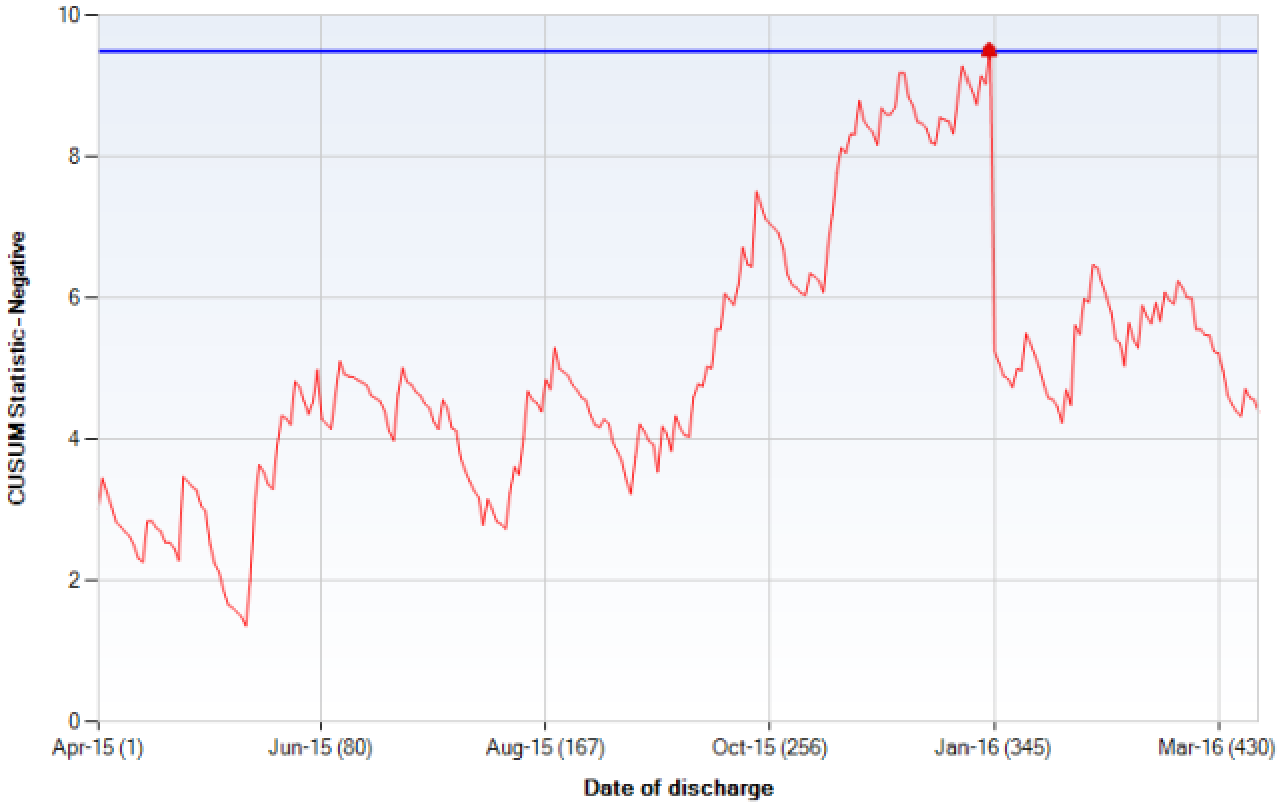

Superspells: 445
First / Last: Apr 2015/Mar 2016
Deaths: 72 (16.2%)
Expected: 47.4 (10.7%)
Observed minus expected: 24.6 (5.5%)
Relative Risk: 151.8 (118.8—191.2)
C-Statistic: 0.77 (Average)
Alerts (X): 1 (Jan-2016)

The probability of a false alarm for this trust in a twelve month period: 0.1%

Appendix 1

DEFINITIONS

The threshold for alerts is set at a high level to ensure an estimated false alarm rate of 0.1% over a 12 month period of monitoring (see discussion of methodology at Appendix 2).

(a) Superspells:

A continuous period of care for an individual, which might include transfers from one hospital to another, and therefore more than one admission (spell). Two different numbers are shown in brackets, the first of which refers to inpatients, the second to daycases; the latter are assumed to have a zero risk of mortality

(b) First / Last:

Time period covered by the graph - the most recent 12 months for which we have data

(c) Deaths (x%) :

Number of deaths and percentage of superspells ending in death

(d) Expected (x%):

Risk adjusted expected number of deaths based on England average mortality

(e) Observed minus expected:

(c) – (d)

(f) Relative Risk:

This is calculated by dividing the observed mortality rate by the expected mortality rate and multiplying by 100. A relative risk of 100 would mean the trust was at the England average. The range refers to 95% confidence intervals

(g) C-Statistic:

The c-statistic is a measure of how well the risk model used for a given diagnosis or procedure predicts the outcome. It is a score between 0.5 and 1.0, where the former represents a predictive power no better than using the crude national rate and perfect predictive power (the model fully explains all variation in outcome). The score that you see is the average value for all the patients in the analysis which we have banded as follows:

0.5 to <0.6 = Very Poor
0.6 to <0.7 = Poor
0.7 to <0.8 = Fair
0.8 to <0.9 = Good
0.9+ = Excellent

(h) Alerts (x):

Number of alerts over the last 12 months and when they occurred

(i) The probability of a false alarm for this trust in a twelve month period:

This is derived from simulation in which 1000s of artificial hospitals, all with the same expected death rate for this diagnosis or procedure group and with the same number of admissions as the annual average for this trust, were monitored for five years. The proportion of them that had an alert in the last year of monitoring is therefore the false alarm rate for this trust in a twelve month period and is given here.

Appendix 2

NOTE ON METHODOLOGY

The analysis is based on HES data. We have analysed the data, covering all major diagnoses and procedures, and identified instances where a given trust's death rates are at least double the expected mortality rate, based on the national average, in the most recent month for which data are available.

To track mortality rates we use cumulative sum charts (CUSUM) charts, which are widely used for quality control in industry, and increasingly in healthcare. We now explain the technique in brief. We determine for each patient the probability of death based on a number of variables (age, sex, whether they are an elective or emergency admission, any pre-existing co-morbidities, sub-diagnosis/procedure, socio-economic group, number of previous admissions, whether they are receiving palliative care, year of discharge, month of admission and source of admission; this risk adjustment is based on rolling 10 years of national data). Based on this, a risk-based score is determined for each patient of death or survival and the function of the difference between actual outcome versus expected outcome is plotted cumulatively.

An acceptable series of outcomes produces a graph where the cumulative score varies randomly at or around a baseline (each poor outcome, ie death, is compensated for by a larger number of good outcomes), whereas a series of poor outcomes will show the chart sloping upwards. Once the chart reaches a pre-set level an alert will be registered, indicating that there is a significant risk of an unacceptably high death rate (odds ratio = 2). Technically, the clinical group or procedure under scrutiny is then deemed "out of control".

The threshold at which we register an alert is set at a high level to minimise false alarms and maximise the possibility that what we are observing is not merely random variation. Based on a large number of computer simulations, we tailor the threshold to each trust and group so that the false alarm rate is less than 0.1% over the course of a year, based on the expected case-mix adjusted mortality, and the average diagnosis or procedure specific volume of your trust over a 12 month period.

Once an alert is sounded the chart is then automatically re-set at half the alert level. Resetting in this way ensures that improved death rates can be identified (if the alert level were not reset, the unit could continue to trigger alerts, even if its death rate improved significantly). But resetting at half the alert level ensures that continued high death rates will rapidly trigger another alert. Because every individual outcome is tracked in this way CUSUM charts are adept at rapidly identifying changes in death rates; we therefore intend to use this methodology to track whether they improve following an alert. If the death rate remains unacceptable the chart will continue to slope upwards again towards the trigger point, whereas acceptable levels would keep the unit below the trigger threshold.

The methodology has been widely discussed in the scientific literature and in itself is generally accepted as robust (see a sample of references below).

References

Jarman B, Gault S, Alves B, Hider A, Dolan S, Cook A, Hurwitz B, Iezzoni LI. Explaining differences in English hospital death rates using routinely collected data. BMJ 1999; 318:1515

Steiner SH, Cook RJ, Farewell VT, Treasure T. Monitoring surgical performance using risk-adjusted cumulative sum charts. Biostatistics 2000;1(4):441-452.

Cook D, Steiner SH, Cook RJ, Farewell VT, Morton AP. Monitoring the evolutionary process of quality: Risk-adjusted charting to track outcomes in intensive care. Crit Care Med 2003; 31(6): 1676-1682.

Jarman, B, Bottle, A, Aylin, P, Browne, M. Monitoring changes in hospital standardised mortality ratios. BMJ 2005, 330: 329

Marshall EC, Best NG, Bottle A, Aylin P. Statistical issues in the prospective monitoring of health outcomes at multiple units. J R Stat Soc Series A 2004;167(3):541-559.

Bottle A, Aylin P. Intelligent Information: a national system for monitoring clinical performance. Health Services Research 2007 (OnlineEarly Articles); http://tinyurl.com/256tpw

Aylin P; Bottle A; Majeed A. (2007) Use of administrative data or clinical databases as predictors of risk of death in hospital: comparison of models. BMJ 2007;334: 1044
