# Supplementary material for: Investigating the association of alerts from a national mortality surveillance system with subsequent hospital mortality in England: an interrupted time series analysis
Source: BMJ Qual Saf. 2018 May 4;27(12):965–73. doi: 10.1136/bmjqs-2017-007495 (PMC6288695; doi:10.1136/bmjqs-2017-007495)
Supplement: Supplementary data [file bmjqs-2017-007495supp002.pdf]

Supplementary file 2

Table S1: List of procedure and diagnosis groups, with frequency of alerts (analysed), between 01/01/2012 and 31/12/20/13

Description	Frequency
**Diagnosis**	
Acute and unspecified renal failure	7
Acute bronchitis	5
Acute cerebrovascular disease	3
Acute Myocardial Infarction	8
Aortic, peripheral, and visceral artery aneurysms	1
Asthma	0
Biliary tract disease	2
Cardiac dysrhythmias	3
Chronic obstructive pulmonary disease and bronchiectasis	6
Chronic renal failure	3
Chronic ulcer of skin	1
Complication of device, implant or graft	1
Complications of surgical procedures or medical care	2
Coronary atherosclerosis and other heart disease	3
Deficiency and other anaemia	5
Diabetes mellitus with complications	1
Diabetes mellitus without complication	0
Epilepsy, convulsions	0
Fluid and electrolyte disorders	9
Fracture of neck of femur (hip)	4
Gastrointestinal haemorrhage	1
Heart valve disorders	0
Intestinal obstruction without hernia	5
Intracranial injury	7
Liveborn	2
Liver disease, alcohol-related	10
Noninfectious gastroenteritis	0
Other psychoses	2
Peripheral and visceral atherosclerosis	7
Peritonitis and intestinal abscess	2
Pleurisy, pneumothorax, pulmonary collapse	0
Pulmonary heart disease	2
Septicemia (except in labour)	19
Skin and subcutaneous tissue infections	2
Spondylosis, intervertebral disc disorders, other back problems	3
Urinary tract infections	6

Description	Frequency
**Procedures**	
Amputation of leg	1
CABG (isolated first time)	0
CABG (other)	8
Cardiac pacemaker or defibrillator introduced through the vein	1
Clip and coil aneurysms	3
Coronary angioplasty (PTCA)	1
Craniotomy for trauma	2
Excision of colon and/or rectum	0
Excision of lung	1
Head of femur replacement	5
Hip replacement	0
Other femoral bypass	0
Reduction of fracture of bone (upper/lower limb)	1
Reduction of fracture of neck of femur	1
Repair of abdominal aortic aneurysm (AAA)	2
Shunting for hydrocephalus	2
Therapeutic endoscopic procedures on biliary tract	5
Therapeutic endoscopic procedures on lower GI tract	0
Therapeutic endoscopic procedures on upper GI tract	5
Therapeutic operations on jejunum and ileum	1
Transluminal operations on femoral artery	1

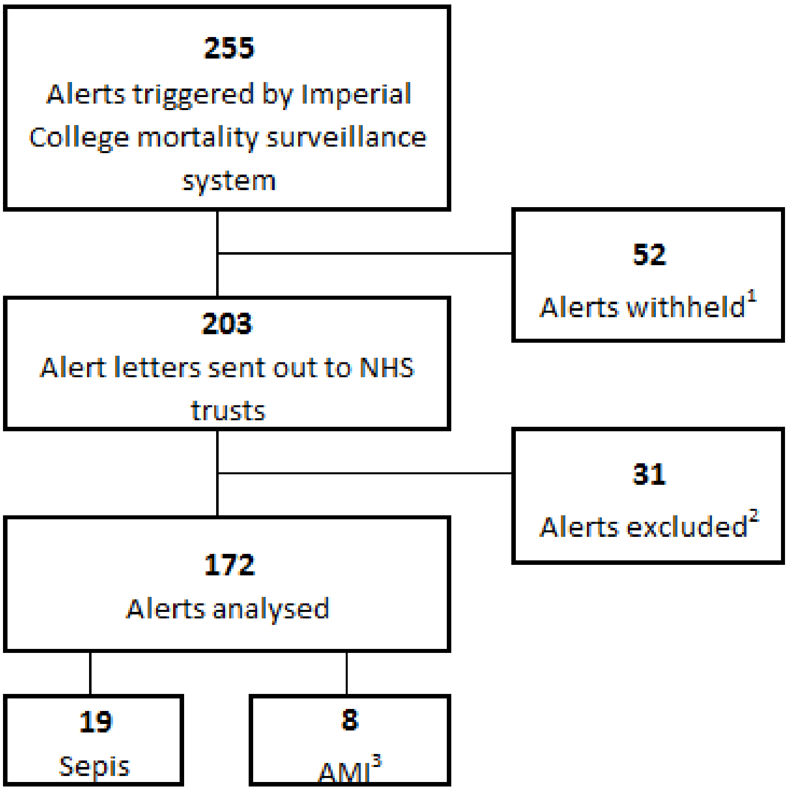

1. Alerts withheld if they a) represent small numbers of deaths (fewer than five expected deaths), or b) they are repeat signals (for a diagnosis/procedure group), within 9 months, for which the trust has already been alerted.
2. Alerts excluded if a) there is insuffiecient trust data to follow up (this can occur when trusts close or merge), or b) if there is a repeat sent alert (for a diagnosis/procedure group) during the 35 months of follow up.
3. Acute myocardial Infarction.

Figure S1: Flow of alerts triggered in 2011 to 2013 and analysed in the study

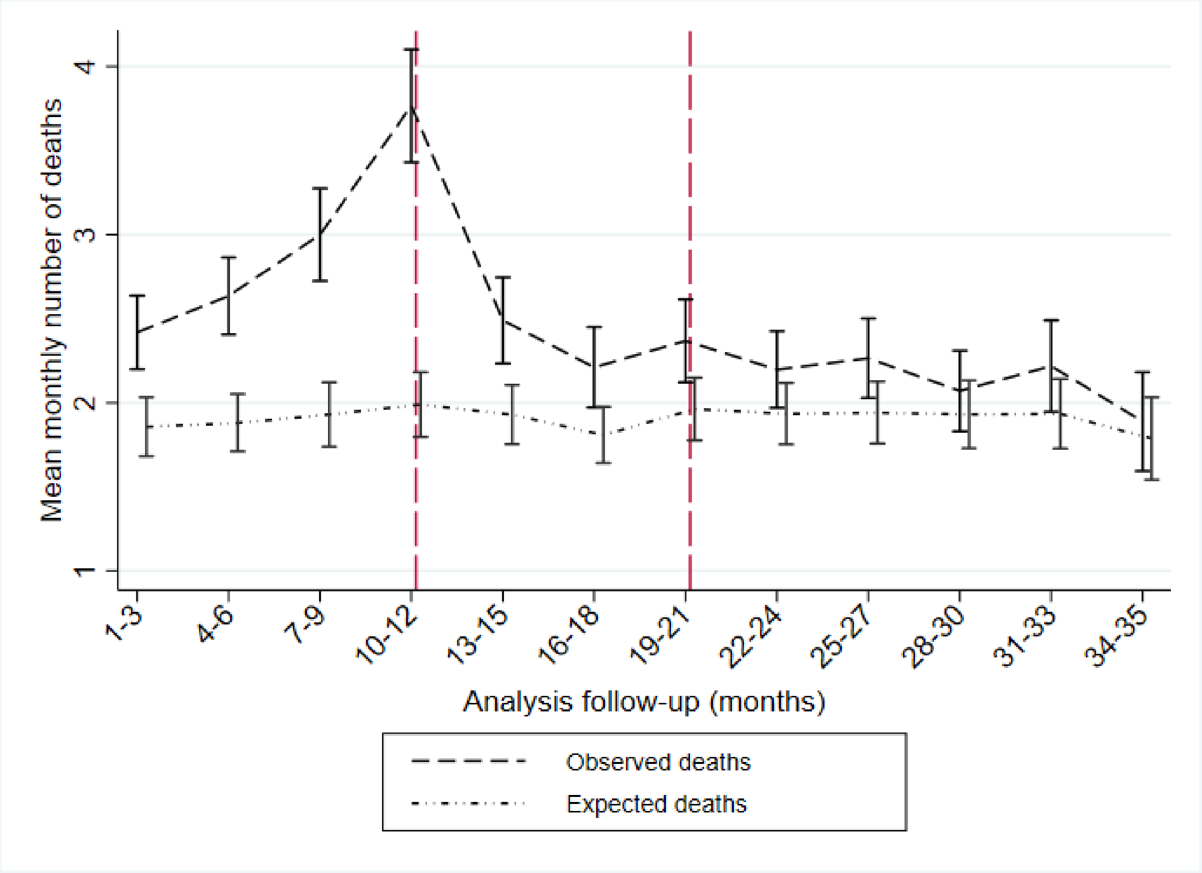

Figure S2: Mean monthly number of observed and expected deaths, with 95% confidence intervals

Mean monthly statistics are calculated from individual trust, diagnosis/procedure group data. Observed and expected number of deaths are in-patient deaths. Expected deaths are estimated using case-mix risk adjustment. The first dropline represents the point at which an alert is triggered. The second dropline represents the end of a 9 months lag, the estimated time for a hospital trust to receive the mortality alert letter, to investigate potential causes and effect change within the hospital setting

Table S2: Descriptive statistics of admissions, deaths, expected deaths and relative risk before and after receipt of an alert letter for sepsis and AMI

	AMI (n=11)		Sepsis (n=19)	
	**Min, Max**	**Median [IQR]**	**Min, Max**	**Median [IQR]**
Admissions				
Counts: Alert letter*	144, 992	377 [331 to 497]	52, 298	171 [112 to 231]
Counts: HES** extract	149, 741	376 [268 to 545]	52, 288	145 [103 to 217]
Absolute Change	-251, 48	-1 [-29 to 5]	-98, 3	-6 [-14 to -1]
Relative Change (%)	-26.5, 9.7	-0.3 [-4.6 to 1.3]	-48.3, 1.7	-3.4 [-11.5 to -0.4]
Deaths (Observed)				
Counts: Alert letter*	22, 99	61 [52 to 78]	29, 107	72 [44 to 82]
Counts: HES** extract	18, 81	60 [45 to 76]	25, 103	46 [34 to 81]
Absolute change	-23, 3	-2 [-7 to 0]	-38, 2	-3 [-12 to 0]
Relative Change (%)	-36.1, 3.8	-2.9 [-18.2 to 0.0]	-60.3, 2.7	-3.7 [-21.1 to 0.0]
Deaths (Expected)***				
Alert letter*	12.4, 74.4	36.9 [34.1 to 52.4]	15.2, 85.7	48.1 [32.8 to 67.2]
HES** extract	12.4, 57.7	36.4 [30.0 to 48.9]	14.1, 69.7	36.8 [21.9 to 57.0]
Absolute change	-17.8, 0.9	-1.2 [-3.6 to 0.2]	-20.0, -0.6	-6.4 [-13.5 to -3.3]
Relative Change (%)	-26.8, 1.8	-4.0 [-10.3 to 0.5]	-47.3, -1.1	-15.6 [-19.8 to -7.1]
Relative risk****				
Alert letter*	133, 177	156 [141 to 167]	118, 191	134 [127 to 150]
HES** extract	133, 173	147 [139 to 166]	109, 205	141 [135 to 149]
Absolute change	-31, 10	1 [-7.9 to 3]	-37, 26	7 [0.3 to 19]
Relative Change (%)	-17, 7	1 [-5.6 to 2]	-25, 18	5 [0.2 to 12]

* Data taken from the sent alert letters covers a year including the month of the alert and 11 months prior.
** Data covering the same time period, trust and diagnosis/procedure as the alert letters, were taken from annual HES extract.
*** The expected number of deaths is derived from a risk adjusted model. The same risk adjustment is used on the annual HES extract as that used when generating mortality alerts.
**** Relative risk is calculated as the observed number deaths divided by the expected number of deaths
